# Contrast-enhanced mammography versus breast MRI in the preoperative evaluation of the nipple-areola complex: data from a real-world setting

**DOI:** 10.1007/s00330-026-12375-0

**Published:** 2026-02-24

**Authors:** Michele Lorenzon, Paola Minichetti, Laura Casotto, Lorenzo Cereser, Chiara Zuiani, Rossano Girometti

**Affiliations:** 1 Institute of Radiology, University Hospital S. Maria della Misericordia, Azienda Sanitaria Universitaria Friuli Centrale (ASUFC), Udine, Italy; 2 Institute of Radiology, Department of Medicine (DMED), University of Udine, Udine, Italy

**Keywords:** Breast cancer, Contrast-enhanced mammography, Magnetic resonance imaging, Nipple-areola complex, Preoperative staging

## Abstract

**Objectives:**

Accurate preoperative assessment of nipple-areolar complex (NAC) involvement is critical for surgical planning in breast cancer patients. While magnetic resonance imaging (MRI) is the established modality for NAC evaluation, contrast-enhanced mammography (CEM) has emerged as a potential alternative. This study aims to compare the diagnostic performance of CEM and MRI in predicting NAC involvement in a real-world setting, using histopathology as the reference standard.

**Materials and methods:**

A retrospective cohort of 195 women with biopsy-proven breast cancer (91 CEM, median age 66 years; 104 MRI, 53 years) underwent preoperative imaging at a single institution. Imaging criteria for NAC involvement were defined. Two radiologists independently evaluated the images, with discordances resolved by consensus. Diagnostic performance was compared between CEM and MRI using Fisher’s exact test. Multivariable logistic regression identified predictors of NAC involvement.

**Results:**

NAC involvement was histologically confirmed in 11.0% (10/91) CEM and 19.2% (20/104) MRI cases (*p* = 0.110). Sensitivity was similar, 60% for CEM and 50% for MRI (*p* = 0.897), with nearly identical specificity (96.3% vs. 96.4%; *p* = 0.709). In situ components were frequently observed in NAC-positive cases (CEM: 90%; MRI: 75%) but were not statistically predictive. Independent predictors included peri-areolar thickening for CEM (OR 26.3; 95% CI 3.9–100; *p* = 0.0007) and abnormal NAC enhancement (OR 14; 95% CI 1.5–133.5; *p* < 0.001) and shorter tumor-to-nipple distance (OR 0.28, 95% CI 0.1–0.6; *p* < 0.001) for MRI.

**Conclusions:**

Notwithstanding a difference in patient age between the CEM and MRI cohorts, which mirrors the different clinical application of the two imaging modalities, CEM demonstrated diagnostic performance comparable to MRI for NAC assessment, offering high specificity and clinical utility in patients unsuitable for MRI. Key imaging features (e.g., peri-areolar thickening, NAC enhancement) aid preoperative decision-making. These findings support CEM as a viable alternative for NAC evaluation, particularly in cases of contraindications to MRI.

**Key Points:**

***Question*** *Preoperative assessment of nipple-areola complex (NAC) involvement in breast cancer impacts surgical decisions. It is unclear whether contrast-enhanced mammography (CEM) may provide diagnostic performance comparable to magnetic resonance imaging (MRI) in assessing NAC involvement.*

***Findings*** *CEM and MRI show comparable diagnostic performance for NAC involvement, with similar sensitivity, specificity, and predictive values.*

***Clinical relevance*** *CEM offers a reliable, accessible alternative to MRI for evaluating NAC involvement, supporting surgical planning in breast cancer patients, especially when MRI is contraindicated or unavailable.*

**Graphical Abstract:**

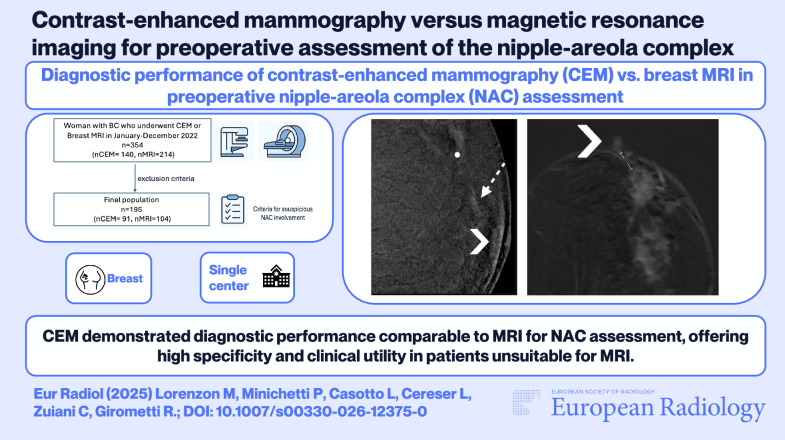

## Introduction

The evolution of surgical techniques for breast cancer (BC) treatment has progressively shifted toward breast-conserving strategies [1–3], highlighting the critical role of accurate locoregional staging and preoperative planning [4]. A pivotal factor in this setting is the evaluation of Nipple-Areola Complex (NAC) involvement, as its infiltration contraindicates nipple-sparing procedures [5]. Imaging techniques have a key role in evaluating NAC involvement, aiding surgical decision-making and optimizing oncological and aesthetic outcomes [6].

Magnetic Resonance Imaging (MRI) is regarded as the most accurate modality for preoperative locoregional staging in BC [7–12], and outperforms mammography and ultrasound in evaluating NAC [6]. According to a recent meta-analysis [6], MRI achieves a pooled sensitivity and specificity of 71% and 94% in assessing NAC involvement, respectively. On the other hand, while breast MRI retains advantages such as superior sensitivity for additional cancer foci [13], contrast-enhanced mammography (CEM) is increasingly used as a reliable alternative for locoregional staging of BC [14, 15], offering optimal diagnostic performance while being a potentially more accessible, faster and well tolerated preoperative procedure, especially in older or MRI unsuitable patients. To confirm a clinically significant role in the locoregional staging of BC, CEM must demonstrate a diagnostic performance comparable to MRI, also in assessing NAC involvement.

However, while previous studies evaluated the accuracy of MRI in the preoperative evaluation of the NAC [16–27], as far as we know, only one study has investigated the accuracy of CEM for the assessment of NAC infiltration [28]. Therefore, it is difficult to understand whether CEM can ensure adequate surgical planning and safe oncological outcomes when NAC is involved.

The aim of this study was to compare the diagnostic performance of CEM and breast MRI in the preoperative assessment of NAC in a real-world setting.

## Materials and methods

### Study population and standard of reference

The study received approval from the Institutional Review Board and was granted a waiver for patient informed consent due to its retrospective design. We retrospectively included patients with biopsy-proven BC who underwent either CEM or MRI at our institution for preoperative assessment between January and December 2022. Indications for preoperative imaging followed the European Society of Breast Cancer Specialists (EUSOMA) criteria [29] or were based on clinical request. The selection between CEM and MRI reflected our institutional policy, in routine clinical practice, of allocating to CEM women aged ≥ 60 years and/or those with relative or absolute contraindication to MRI, reserving MRI to all other patients. The age cutoff of 60 years was chosen as a practical, non-mandatory guideline for the following reasons: (1) Adherence to radiation protection principles by favoring techniques like MRI, that do not use ionizing radiation, in younger patients; (2) The greater ease of execution and patient tolerability of CEM in an older population; (3) The lack of an established, standardized age-based criterion in the published literature [30].

**Fig. 1:**
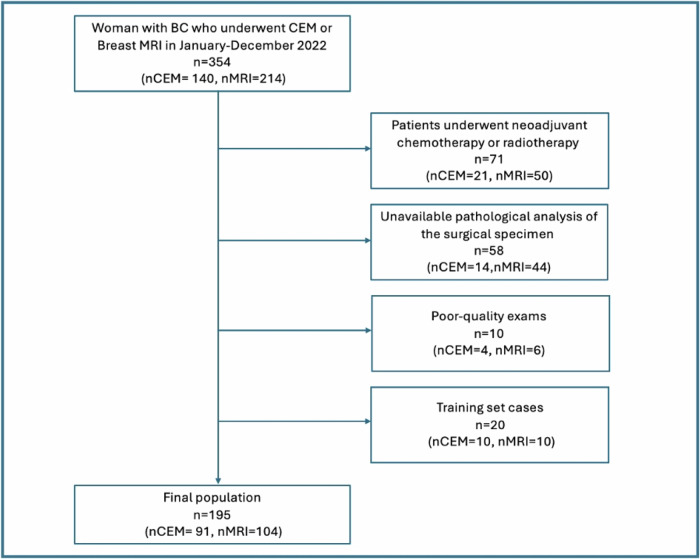
Study flowchart. BC, breast cancer; CEM, contrast-enhanced mammography; MRI, magnetic resonance imaging

After applying the exclusion criteria reported in Fig. 1, the final population consisted of 195 women who underwent CEM or MRI in 91 and 104 cases, respectively.

The reference standard consisted of histopathological evaluation of surgical specimens, performed by one of three board-certified pathologists with 5–25 years of subspecialty experience in breast pathology, in accordance with College of American Pathologists guidelines [31]. Breast cancer histotypes were documented, as well as the presence or absence of intraductal extension. NAC involvement was histologically confirmed when pathological examination of the surgically resected specimen demonstrated the presence within the nipple tissue of either: (1) invasive carcinoma components, (2) ductal carcinoma in situ (DCIS), or (3) Paget’s disease cells. In nipple-sparing mastectomy cases, histopathological evaluation included assessment of both the retroareolar disk and surgical margins [32]. Full histopathological assessment of the NAC was reserved for total or skin-sparing mastectomy specimens, as for central quadrantectomies. For patients treated with quadrantectomy or lumpectomy, negative surgical margins were deemed to infer negative nipple status.

### CEM and MRI technique

CEM was performed with a dedicated system (Selenia Dimensions 3D, Hologic) following intravenous injection of 1.2 mL/Kg of iodinate contrast medium (Iobitridol 350 mg/mL, Guerbet) and a 20 mL bolus of saline solution. Injection was performed under remote control (Accutron CT-D, Medtron) at an injection rate of 3 mL/s. The protocol included the acquisition of right-sided cranio-caudal (CC) and medio-lateral oblique (MLO) views 2 min after contrast administration, immediately followed by left-sided CC and MLO views. Seven minutes post-contrast injection, CC and/or MLO views of the affected side were obtained, depending on the decision of the attending radiologist.

MRI examinations were performed on one of two 1.5 T magnets (Magnetom Aera or Magnetom Avanto, Siemens Medical Solutions), with a bilateral 16-channel coil and the patient in prone position. Standard breast MRI protocol included diffusion-weighted imaging (DWI), T2-weighted imaging and T1-weighted, non-fat-saturated dynamic contrast-enhanced imaging (DCE) with and without digital subtraction, as detailed in Table 1. A contrast dose of 0.1 mmol/kg gadoteridol (Prohance, Bracco Imaging) was administered at an injection rate of 2 mL/s, followed by a 20 mL bolus of saline solution. Injection was performed under remote control.

**Table 1:** Acquisition parameters of the MRI protocol

	DWI	DCE	T2w
Sequence	Single-shot EPI	3D FLASH	TSE
Plane	Transverse	Transverse	Transverse
TR/TE (msec)	5700/60	9/4.76	4900/76
Field of view (mm × mm)	350 × 171.6	350 × 350	340 × 340
Matrix (pixels × pixels)	102 × 208	492 × 512	384 × 384
Slice thickness (mm)	4	2	3
Interslice gap (mm)	1	-	0.8
In-plane resolution (mm)	1.7 × 1.7	0.7 × 0.7	0.9 × 0.9
Number of slices	32	80	43
b-values (s/mm^2^)	0, 800	-	-
Number of excitation	2 for b = 0, 4 for b = 800	1	2
Fat saturation	SPAIR	None	None
Parallel imaging (algorithm/acceleration factor)	GRAPPA/2	GRAPPA/2	GRAPPA/2
Acquisition time	1.43 min	1 unenhanced phase followed by 5 post-contrast phases, 1.32 min each	4.41

*DCE* dynamic contrast-enhanced imaging, *EPI* echoplanar imaging, *FLASH* fast long angle shot, *GRAPPA* generalized autocalibrating partial parallel acquisition, *TE* echo time, *TSE* turbo spin echo, *TR* repetition time, *SPAIR* spectrally adiabatic inversion recovery

### Image analysis

An independent study coordinator, not involved in the image interpretation process, arranged dedicated reading sessions with two radiologists who met EUSOMA and European Cancer Concord expert criteria [33]. Reader 1 (R1) and reader 2 (R2) had 15 and 4 years of continued clinical activity in breast imaging, respectively. Both readers were blinded to clinical, histopathological or follow-up information, except for the knowledge of BC presence and location of the index lesion (IL), defined as the previously confirmed tumor prompting locoregional staging.

Study readings were preceded by a training session during which R1 and R2 reviewed 10 CEM and 10 MRI exams, randomly selected by the study coordinator. The training phase aimed to standardize the evaluation based on normal findings (e.g., linear symmetrical or asymmetrical enhancement [34]) and the suspicion criteria listed below. During study reading sessions, R1 and R2 were asked to independently review randomly presented CEM or MRI exams, focusing on NAC. CEM included both low-energy (LE) and recombined (REC) images, while MRI included all the images and maximum intensity projections reconstructions obtained from the first post-contrast DCE sequence. NAC involvement was assessed based on one or more of the following criteria: (1) nipple retraction and invasion; (2) abnormal enhancement of NAC, particularly when compared with the contralateral NAC; (3) tumor nipple enhancement (TNE), i.e., presence of enhancement between tumor and nipple base [35]; (4) peri-areolar skin thickening, i.e., thickening of the peri-areolar zone; (5) abnormal morphology or asymmetry of the NAC; (6) a tumor-to-nipple (TTN) distance ≤ 10 mm, measured from the nearest tumor margin to the base of the nipple. In cases of multifocal or multicentric disease, the shortest distance between any tumor focus and the nipple base was considered [7]. Moreover, contrast-enhancement pattern (mass vs. non-mass), tumor size, and, for CEM examinations, associated microcalcifications were also assessed.

Peri-areolar thickening may, in some cases, reflect reactive or inflammatory edema rather than true neoplastic infiltration. In our study, the distinction between these conditions was based on a combined evaluation of morphology, enhancement pattern, and T2-weighted signal characteristics (on MRI). Edema was defined as diffuse and symmetric thickening with preservation of the smooth contour and layered structure of the NAC, showing homogeneous hyperintensity on T2-weighted sequences on MRI and minimal or no enhancement on CEM. True NAC infiltration, instead, was identified when architectural distortion, irregular or nodular enhancement, and loss of the normal dermal–epidermal interface were observed, often contiguous with the primary lesion or involved ducts. These radiologic criteria are consistent with previously reported features in breast MRI literature [12, 36].

The readers performed independent evaluations, exercising complete discretion in subjectively integrating the aforementioned imaging features to formulate a binary determination regarding NAC involvement (present/absent). As a general guideline, it was suggested to consider that the presence of ≥ 2 criteria increased the likelihood of NAC involvement.

Ancillary features such as location and IL enhancement, breast density category according to the Breast Imaging Reporting and Data System (BI-RADS) [37, 38] and Background Parenchymal Enhancement (BPE) were also recorded. For the purpose of analysis, BI-RADS density categories A and B were considered as “non-dense” while categories C and D were regarded as “dense” either directly on CEM or based on previous mammography examinations in the MRI group.

After independent readings, discordant cases were resolved through consensus to establish a single, unified set of CEM and MRI readings for analysis.

### Statistical analysis

We used descriptive statistics to report the main clinical and imaging variables. Given that the Shapiro–Wilk test indicated a non-normal distribution of continuous variables, they were summarized with median values and interquartile range (IQR). Comparisons between the CEM and MRI groups were performed with the Mann–Whitney U test. Categorical variables were compared with the chi-square test. Relevant proportions were complemented with 95% confidence intervals (95% CI).

Given the inherently different populations, it was not feasible to apply a propensity score matching approach. Therefore, we assessed whether the main patient-related characteristics associated with referral to CEM or MRI—namely age and breast density—were significantly different between the two groups (Table 2).

**Table 2:** Comparison of main clinical, histopathological, and imaging features between women who underwent contrast-enhanced mammography (CEM) and breast magnetic resonance imaging (MRI)

		CEM (*n* = 91)	MRI (*n* = 104)	*p*-value
Clinical				
Age median (IQR)		66 (59–72)	53 (48–62.3)	< 0.001
Breast density according to BI-RADS *n* (%)	Non-dense Dense	44 (48.4) 47 (51.6)	27 (26) 77 (74)	0.284
Histological				
Histotype *n* (%)	IBC-NST DCIS ILC Other	61 (67.03) 14 (15.38) 13 (14.29) 3 (3.3)	63 (60.6) 19 (18.3) 18 (17.31) 4 (3.85)	0.904
Presence of “in situ” component *n* (%)	Yes No	59 (64.8) 32 (35.2)	76 (73.1) 28 (26.9)	0.945
Immunohistochemical profile *n* (%)	Luminal A Luminal B/HER2- Luminal B/HER2+ HER2-enriched Triple-negative	49 (53.9) 27 (29.7) 10 (11.0) 2 (2.2) 3 (3.3)	72 (69.2) 18 (17.3) 4 (3.9) 0 (0) 10 (9.6)	0.333
NAC positivity *n* (%)		10 (11)	20 (19.2)	0.110
Imaging				
BPE *n* (%)	Minimal Mild Moderate Marked	18 (19.8) 42 (46.2) 28 (30.8) 3 (3.3)	2 (1.9) 26 (25) 58 (55.8) 18 (17.3)	0.063
Type of lesion enhancement *n* (%)	Mass Non-mass Mass + Non-mass	75 (82.4) 5 (5.5) 11 (12.1)	84 (80.8) 15 (14.4) 5 (4.8)	0.386
Lesion dimension (mm) *n* (%)	< 10 10–20 > 20	16 (17.6) 34 (37.4) 41 (45.1)	8 (17.3) 38 (36.5) 48 (46.2)	0.215

*p*-values for comparisons were obtained with the Mann–Whitney U test for patient age and with χ^2^ test for all other variables

“Other”: 1 Paget, 2 ca. mucinous in CEM group and 2 DCIS + Paget, 1 DCIS + lobular carcinoma in situ, 1 ca. mucinous in MRI group

*BI-RADS* Breast Imaging Reporting and Data System, *IBC-NST* invasive breast carcinoma of non-special type, *DCIS* ductal carcinoma in situ, *ILC* invasive lobular carcinoma, *BPE* background parenchymal enhancement, *NAC* Nipple-areolar complex

By matching the radiologists’ readings with the reference standard, we calculated the sensitivity, specificity, positive (PPV), and negative predictive value (NPV) for NAC involvement. We used Fisher’s exact test to compare the diagnostic performance of CEM versus MRI. Inter-reader agreement for the binary CEM and MRI rating was quantified using Gwet’s AC1 as the primary statistic, given the known instability of Cohen’s κ under marked prevalence imbalance, percent agreement and Cohen’s κ were also reported for transparency [39–41].

Logistic regression analyses were applied to identify independent predictors of NAC involvement among clinical and imaging variables. A stepwise selection method was employed, including variables with a significance threshold of *p* < 0.05. Two separate models were developed: one for CEM and one for MRI. The clinical variables included in both models were as follows: age (divided into quartiles according to its distribution within the study cohort), histological type of breast cancer (divided into IBC-NST, ILC, DCIS and other), in situ component (present or absent) and immunohistochemical profile (divided into Luminal A, Luminal B/Her2-, Luminal B/Her2+, Triple-negative and Her2+). In addition, the following imaging-specific variables were included in each model: breast density (Dense vs. Non-dense breasts), BPE enhancement (divided into low, mild, moderate, marked), nipple retraction and invasion, abnormal enhancement of NAC (present or absent), TNE, peri-areolar skin thickening, abnormal morphology or asymmetry of the NAC, TTN distance (divided into quartiles according to its distribution in the cohort), contrast-enhancement pattern (mass vs. non-mass), tumor size, and, for CEM examinations, associated microcalcifications.

As a second step, we fitted an additional multivariable model to test whether the association of age and breast density with NAC involvement differed according to the imaging modality (CEM versus MRI). This model included the imaging modality as a categorical variable (CEM vs. MRI), as well as age, breast density, and their interaction terms with the imaging modality (calculated as the product of each variable and the modality indicator) [42].

Analyses were performed using commercially available software (MedCalc® Statistical Software version 23.1.3, MedCalc Software Ltd.) and Stata/BE 19.5. Alfa level was 0.05.

## Results

### Study population and NAC involvement

Clinical features and imaging findings unrelated to NAC involvement are summarized in Table 2. Specifically, the median patient age (IQR) was 66 (59–72) years in the CEM group and 53 (48–62.3) years in the MRI group (*p* < 0.01, Mann–Whitney U test). The distribution of breast density was not significantly different (*p* = 0.284, chi-square test) in the two cohorts.

At pathological examination, NAC infiltration was observed in 10 of 91 women in the CEM group (11.0%; 95% CI: 5.4–19.2) and in 20 of 104 women in the MRI group (19.2%; 95% CI: 12.1–28.1), a difference that did not reach statistical significance (*p* = 0.110). Among patients with NAC infiltration, both in the CEM and MRI groups, ductal carcinoma in situ (DCIS) was the prevalent histological subtype.

In the MRI cohort, pure DCIS was identified in 10 cases. In situ components were present in the majority of cases (15 out of 20), including the abovementioned pure DCIS (*n* = 10), but also invasive breast carcinoma of no special type (IBC-NST) (*n* = 1), and other subtypes (*n* = 4). Notably, three cases of IBC-NST and two cases of invasive lobular carcinoma (ILC) lacked any in situ component.

Similarly, in the CEM group, pure DCIS was the most frequently observed subtype associated with NAC infiltration, identified in 5 cases. Additional histological subtypes included IBC-NST (*n* = 2) and ILC (*n* = 2), both of which retained in situ components. A single case classified as ‘other’ did not display any in situ component. Therefore, 9 out of 10 cases with NAC infiltration in the CEM group exhibited an in situ component. These results are summarized in Supplementary Table 1.

However, the difference in terms of in situ component prevalence between patients with and without nipple involvement, both in the CEM group and the MRI group, was not statistically significant.

In all cases where the nipple was surgically preserved, the surgical margins and the retroareolar disk (when available) were histologically negative for tumor involvement.

**Fig. 2:**
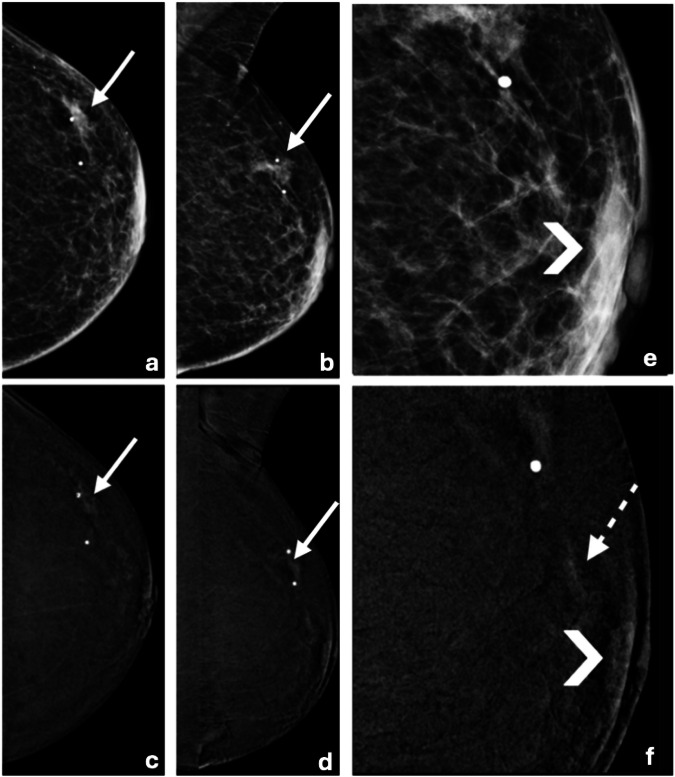
Seventy-four-year-old woman with grade 2 invasive lobular carcinoma (ILC) with an in situ component, undergoing preoperative contrast-enhanced mammography (CEM). The index lesion (arrows) was identified as a mass lesion in the upper-outer quadrant of the left breast in the low-energy cranio-caudal (CC) (**a**), medio-lateral oblique (MLO) views (**b**) and recombined CC (**c**) and MLO (**d**) views. Both readers identified four criteria for suspicious NAC involvement: (i) peri-areolar skin thickening (arrowheads) (**e**, **f**), (ii) tumor nipple enhancement (TNE), characterized by a discontinuous non-mass enhancement between the tumor and nipple base (dashed arrow) (**f**), (iii) abnormal enhancement of the left NAC (**e**, **f**), and (iv) nipple retraction (**e**, **f**). The tumor-to-nipple (TTN) distance was > 10 mm. Post-mastectomy histopathology confirmed grade 2 ILC with an in situ component and NAC involvement

**Fig. 3:**
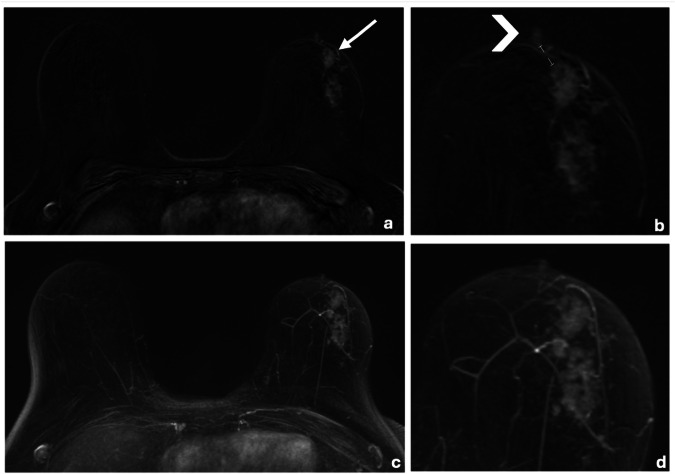
44-year-old woman with grade 3 invasive breast carcinoma of no special type (IBC-NST) with in situ component, undergoing preoperative breast MRI. The tumor was identified as multiple contiguous mass lesions located in the retroareolar region of the left breast (arrow) in the second post-contrast subtracted T1-weighted sequence (**a**). Three criteria suggestive of nipple-areolar complex (NAC) involvement were identified by both readers: (i) abnormal enhancement of the left NAC relative to the contralateral NAC (arrowheads) (**b**), (ii) a tumor-to-nipple (TTN) distance ≤ 10 mm (between calipers) (**b**) and (iii) nipple retraction (**b**). Contrast-enhanced T1-weighted MIP images confirmed and highlighted the described findings (**c**, **d**). Final surgical pathology examination after total mastectomy and axillary dissection confirmed multicentric grade 3 IBC-NST with an in situ component and NAC involvement

Suspicion of NAC infiltration was raised in 9 out of 91 CEM examinations (9.9%; 95% CI: 4.5–18.8) and in 13 out of 104 MRI examinations (12.5%; 95% CI: 6.7–21.4). Figures 2 and 3 demonstrate representative cases of confirmed NAC involvement.

### Diagnostic performance of CEM and MRI, inter-reader agreement and predictive variables

**Fig. 4:**
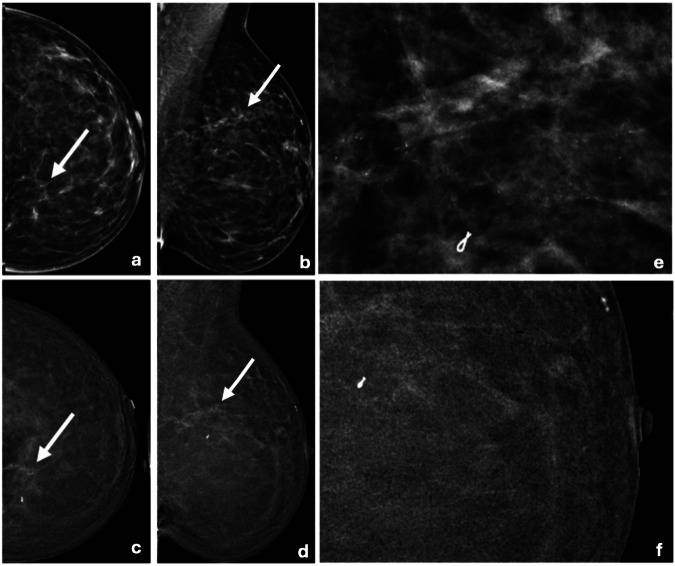
Preoperative contrast-enhanced mammography (CEM) in a 62-year-old woman presenting with grade 3 ductal carcinoma in situ (DCIS). This lesion was detected in the inner quadrants of the left breast (arrows) on low-energy cranio-caudal (CC) (**a**), medio-lateral oblique (MLO) (**b**) and magnification (**e**) views as a 3 cm cluster of fine pleomorphic microcalcifications with a regional distribution, and a clip was placed after tomobiopsy procedure. After contrast administration, the lesion appeared as a regional, heterogeneous, non-mass lesion on recombined cranio-caudal (CC) (**c**) and medio-lateral oblique (MLO) (**d**) views. Detailed evaluation of the NAC shows no suspicious criteria for NAC involvement (**f**). Following quadrantectomy and axillary lymph node dissection, the presence of positive surgical margins mandated completion mastectomy. Definitive histopathological examination revealed grade 3 DCIS with histologically confirmed NAC involvement

**Fig. 5:**
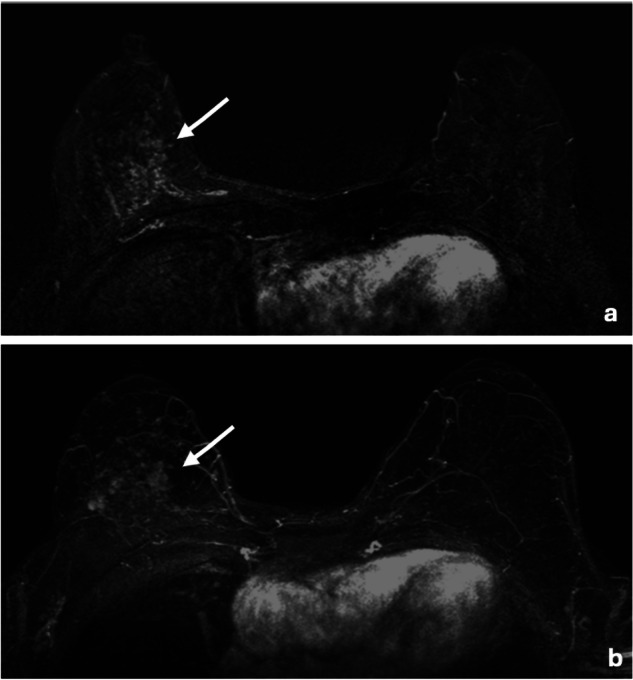
Fifty-three-year-old woman with grade 3 ductal carcinoma in situ (DCIS) undergoing preoperative MRI. The tumor was identified in the retroareolar region of the right breast (arrows), and appears as regional, heterogeneous non-mass lesions in the second post-contrast subtracted T1-weighted (**a**) and contrast-enhanced T1-weighted MIP (**b**) sequences. Both readers found no suspicious criteria for NAC involvement. Post-mastectomy histopathology confirmed grade 3 DCIS with NAC involvement. NAC, nipple-areolar complex

Table 3 summarizes the diagnostic performance of CEM and MRI in assessing NAC infiltration with reported TP/TN/FP and FN (Figs. 4 and 5). No significant differences were observed between the two modalities in terms of sensitivity (CEM 60%, 95% CI 26–87%, MRI 50% 95% CI 27–73%, difference 10%, *p* = 0.897) or specificity (CEM 96.3%, 95% CI 89–99%, MRI 96.4%, 95% CI 83–99%, difference −0.1, *p* = 0.709).

**Table 3:** Diagnostic performance comparison between CEM and MRI in detecting NAC infiltration

Diagnostic performance	CEM	MRI
TP/TN/FP/FN (*n*)	6/78/3/4	10/81/3/10
Sensitivity % (95% CI)	60% (26–87%)	50% (27–73%)
Specificity % (95% CI)	96.3% (89–99%)	96.4% (83–99%)
PPV % (95% CI)	66.7% (37–87%)	76.9% (50–92%)
NPV % (95% CI)	95.1% (90–97%)	89% (84–93%)
Accuracy % (95% CI)	92.3% (84–96%)	87.5% (80–93%)

*TP* true positive, *TN* true negative, *FP* false positive, *FN* false negative, *PPV* positive predictive value, *NPV* negative predictive value, *95% CI* confidence interval, *CEM* contrast-enhanced mammography

The inter-reader agreement for suspicious NAC involvement was almost perfect for both modalities. For CEM, percent agreement was 92.3% (84/91) and Gwet’s AC1 = 0.905 (95% CI 0.832–0.979), indicating near-perfect agreement; Cohen’s κ = 0.589 (95% CI 0.308–0.869). For MRI, percent agreement was 94.2% (98/104), Gwet’s AC1 = 0.926 (95% CI 0.865–0.988), and Cohen’s κ = 0.736 (95% CI 0.532–0.940). The inter-reader agreement results for suspicious NAC involvement are shown in Table 4.

**Table 4:** Inter-reader agreement results for suspicious NAC involvement in CEM and MRI

		PA	Gwet’s AC1	95% CI	Interpretation	Cohen’s Kappa	95% CI	Interpretation
CEM	R1	0.92	0.91	0.83–0.98	Almost perfect	0.59	0.31–0.87	Moderate
	R2							
MRI	R1	0.94	0.93	0.86–0.99	Almost perfect	0.74	0.53–0.94	Substantial
	R2							

*R1* reader 1, *R2* reader 2, *PA* percent agreement, *95% CI* 95% confidence interval, *CEM* contrast-enhanced mammography, *NAC* Nipple-areolar complex

Table 5 refers to the results of the logistic regression analysis. On multivariable analysis, the independent predictors of NAC involvement were peri-areolar thickening in the CEM model, NAC enhancement and TTN distance in the MRI model. In the latter model, a greater TTN distance was associated with a reduced likelihood of NAC involvement, indicating a protective effect.

**Table 5:** Results of logistic regression for the prediction of NAC involvement in CEM and MRI

CEM model				
	Univariable analysis		Multivariable analysis	
	Prevalence of NAC involvement (%)	*p*-value	OR (95% CI)	*p*-value
Age (range in years)		0.5587	-	-
First quartile (47–61)	4/24 (16.7)			
Second quartile (61–66.5)	2/22 (9.1)			
Third quartile (66.5–73)	1/23 (4.4)			
Fourth quartile (73–84.6)	3/22 (13.6)			
Breast density according to BI-RADS		0.2290	-	-
Dense breasts	7/47 (14.9)			
Non-dense breasts	3/44 (6.8)			
Histological type of breast cancer		0.5554	-	-
IBC-NST	6/61 (9.8)			
ILC	2/13 (15.4)			
DCIS	1/14 (7.1)			
Other	1/3 (33.3)			
Presence of “in situ” component		0.2898	-	-
Yes	8/59 (13.6)			
No	2/32 (6.3)			
Immunohistochemical profile		0.9002	-	-
Luminal A	5/49 (10.2)			
Luminal B/Her2-	4/27 (14.8)			
Luminal B/Her2+	1/10 (10)			
Triple-negative	0/3 (0)			
Her2+	0/2 (0)			
BPE enhancement		0.2508	-	-
Low	0/18 (0)			
Mild	5/42 (11.9)			
Moderate	4/28 (14.3)			
Marked	1/3 (33.3)			
Abnormal NAC morphology		0.2108	-	-
Yes	1/3 (33.3)			
No	9/88 (10.2)			
PA thickening		< 0.0001	26.3 (3.9–100)	0.0007
Yes	4/6 (66.7)			
No	6/85 (7.1)			
NAC retraction		0.0001	-	-
Yes	2/2 (100)			
No	8/89 (8.9)			
NAC enhancement		0.0001	-	-
Yes	2/2 (100)			
No	8/89 (8.9)			
TTN distance (mm)		< 0.0001	-	-
< 10	7/87 (8)			
≥ 10	3/4 (75)			
TNE		0.0112	-	-
Yes	2/4 (50.0)			
No	8/87 (9.2)			
Tumor dimension (mm)		0.4722	-	-
< 10	2/16 (12.5)			
10–20	2/34 (5.9)			
> 20	6/41 (14.6)			
Tumor enhancement		0.0140	-	-
Mass	0/5 (0)			
Non-mass	6/75 (8)			
Mass + non-mass	4/11 (36.3)			
Microcalcification		0.4185	-	-
Yes	8/80 (10)			
No	2/11 (18.2)			

MRI model				
Age		0.6117	-	-
First quartile (29–48)	4/31 (12.9)			
Second quartile (48–53)	4/22 (18.2)			
Third quartile (53–62.25)	5/25 (20)			
Fourth quartile (62.25–79)	7/26 (26.9)			
Breast density according to BI-RADS		0.5007	-	-
Dense breasts	16/77 (20.8)			
Non-dense breasts	4/27 (14.8)			
Histological type of breast cancer		0.1922	-	-
IBC-NST	8/63 (12.7)			
CLI	5/19 (26.3)			
DCIS	6/18 (33.3)			
Other	1/4 (25)			
In situ component		0.1831	-	-
Yes	3/28 (10.7)			
No	17/76 (22.4)			
Immunohistochemical profile		0.5724	-	-
Luminal A	16/72 (22.2)			
Luminal B/Her2-	3/18 (16.7)			
Luminal B/Her2+	0/4 (0)			
Triple-negative	1/10 (10)			
Her2+	0/0 (0)			
BPE		0.555	-	-
Low	1/2 (50)			
Mild	6/26 (23.1)			
Moderate	9/58 (15.5)			
Marked	4/18 (22.2)			
Abnormal NAC morphology		0.0003	-	-
Yes	17/101 (16.8)			
No	3/3 (100)			
PA thickening		0.0041	-	-
Yes	17/100 (17)			
No	3/4 (75)			
NAC retraction		0.0025	-	-
Yes	16/98 (16.3)			
No	4/6 (66.7)			
NAC enhancement		< 0.0001	14 (1.5–133.5)	-
Yes	13/96 (13.5)			
No	7/8 (87.5)			
TTN distance (mm)		< 0.0001	0.28 (0.1–0.6)	-
< 10	13/94 (13.8)			
≥ 10	7/10 (70)			
TNE		0.0002	-	-
Yes	14/95 (14.7)			
No	6/9 (66.7)			
Tumor dimension (mm)		0.4902	-	-
< 10	4/18 (22.2)			
10–20	5/38 (13.2)			
> 20	11/48 (22.9)			
Tumor enhancement		0.0575	-	-
Mass	3/15 (20)			
Non-mass	14/84 (16.7)			
Mass + non-mass	3/5 (60)			

*OR* odds ratio*, 95% CI* confidence interval, *BI-RADS* Breast Imaging Reporting and Data System, *IBC-NST* invasive breast carcinoma of non-special type, *DCIS* ductal carcinoma in situ, *ILC* invasive lobular carcinoma, *BPE* background parenchymal enhancement, *PA* peri-areolar, *NAC* nipple-areola complex, *TTN* tumor-to-nipple, *TNE* tumor nipple enhancement

No significant interactions between patient-related variables and the imaging modalities were found in predicting NAC involvement (*p* > 0.05, Supplementary Table 2).

## Discussion

In our series, in the setting of locoregional staging, CEM and MRI provide comparable diagnostic performance in assessing NAC involvement, with no statistically significant differences in sensitivity (60% vs. 50%) and comparable NPV (95.1% vs. 89%). Specificity is nearly identical and very high for both modalities (96.3% for CEM and 96.4 for MRI), suggesting that when either modality raises suspicion of NAC infiltration, there is a strong likelihood of true pathological NAC infiltration.

These findings confirm CEM as a reliable and potentially more accessible alternative to MRI in local staging of BC, filling the knowledge gap with previous studies, which established the diagnostic performance of MRI for NAC evaluation [6, 16–27, 35] and supporting the clinical use of CEM in preoperative staging when MRI is contraindicated, unavailable or unsuitable.

Notably, the sensitivity of MRI in our study is lower than that reported in prior MRI-focused studies, where it has been shown to vary considerably between 60.5% and 90–100% [10, 16–27, 35]. This discrepancy may be attributed to differences in tumor characteristics of NAC-involved specimens compared to those reported in previous studies. Particularly, in two previous studies reporting higher MRI sensitivity [10, 35], the prevalence of invasive tumors with direct nipple invasion was higher (respectively 86% and 82%). Our cohort includes fewer invasive tumors with direct nipple involvement, and, in both imaging groups, ductal carcinoma in situ (DCIS) is the most frequent histological subtype associated with NAC infiltration. In particular, in our case series, the presence of in situ components was frequently observed in patients with nipple involvement (15 out of 20 cases—75%—in the MRI group; 9 out of 10 cases—90%—in the CEM group). However, statistical analysis showed that this finding was not predictive of nipple involvement. Certainly, when in situ components are present in the main lesion, it is important to consider that this type of disease may exhibit very subtle contrast-enhancing features [18, 43].

Our data also confirm that NAC involvement is not uncommon. The observed frequency of NAC involvement is 11.0% in the CEM cohort and 19.2% in the MRI group, consistent with published rates ranging from 5.6% to 35.5% in the literature [35]. The higher rate in the MRI group may reflect the younger age of these patients, consistent with the more aggressive tumor biology typically seen in younger women (higher-grade and hormone receptor-negative tumors, greater prevalence of genetic mutations and basal-like subtypes) [44–48].

Among the imaging criteria assessed, signs more frequently associated with NAC infiltration in both imaging modalities are abnormal NAC enhancement, peri-areolar skin thickening, TNE and nipple retraction. These findings are already well documented in MRI literature [11, 16, 19, 35] and are similarly applicable to CEM in our study.

Multivariable analysis reveals distinct predictors of NAC involvement for each modality, reflecting their inherent imaging differences. In the CEM model, peri-areolar thickening emerges as the strongest independent predictor, whereas in the MRI model, NAC enhancement and TTN distance are significantly associated with NAC infiltration. Interestingly, a greater TTN distance is associated with a reduced likelihood of NAC involvement, suggesting a protective effect as long as the distance between the tumor and NAC increases, as expected. This supports the clinical relevance of TTN measurements as a criterion to guide surgical decision-making, and in particular, the protective effect of increasing TTN distance. A 10 mm threshold was adopted a priori, consistent with recent literature supporting its oncologic safety [49]. Notably, Cozzi et al [28] recently reported a 9.5-mm cutoff on CEM as the best predictor of pathologic nipple involvement, further supporting our approach.

The high inter-reader agreement observed for both modalities further supports the reproducibility of NAC assessment, confirming that the diagnostic criteria applied for CEM and MRI are consistent and reliable across readers.

Despite promising results, the study presents some limitations.

First, the study population groups are inherently different as stratified according to institutional criteria for routine clinical practice allocation. This structured approach results in a significant difference in median patient age between the two groups (*p* < 0.01). Advancing age is usually associated with reduced tumor aggressiveness, mammographic density, and background parenchymal enhancement; therefore, a specific analysis was performed to assess whether this difference could introduce a bias in our study. As reported in the results, while the distribution of breast density is not significantly different, patients’ age is, in fact, significantly greater in the CEM group versus the MRI group. However, in the multivariable model, no significant interactions between patient-related variables and the imaging modalities have been found in predicting NAC involvement (*p* > 0.05), thus suggesting that, although inherently different, age is uninfluential in assessing NAC involvement with either CEM or MRI, and in turn, it does not reasonably represent a bias in our study. Moreover, it is also recognized that the use of CEM is increasing, particularly in high-workload academic settings [30], and its adoption is reasonably expected to grow, especially among older patients (despite the current lack of formal guidelines). Therefore, the age difference between the two patient cohorts can also be interpreted as a finding that reflects, and will probably increasingly reflect, real-world clinical practice. From this perspective, the absence of significant differences in diagnostic performance between the two modalities may serve as evidence that CEM and MRI yield comparable results in slightly different patient populations, thereby ensuring equitable treatment outcomes.

Second, some imaging features, such as NAC retraction, had a very low prevalence among NAC-positive cases. This limited frequency may have introduced instability in the corresponding odds ratios of the logistic regression models, a known issue in analyses with sparse data. Moreover, the relatively small number of NAC-positive cases may limit the robustness of sensitivity estimates and regression analyses. However, we believe that a post hoc power calculation would not add substantial information, as the low prevalence of NAC involvement reflects the expected incidence reported in previous studies [27].

Third, the absence of a standardized lexicon for NAC assessment in CEM and subjective assessment of imaging findings may have introduced interpretative variability.

Future prospective studies with matched populations and standardized imaging criteria are needed to validate these results and refine diagnostic algorithms.

## Conclusion

In conclusion, notwithstanding a difference in patient age between our CEM and MRI cohorts, which mirrors the different clinical use of the two imaging modalities, CEM and MRI show comparable diagnostic performance in the assessment of NAC involvement in breast cancer patients, suggesting their usefulness to guide surgical planning for NAC-sparing procedures. Given the high specificity, great accessibility, fast acquisition time, favorable tolerability, and simple interpretation, CEM may represent a clinically viable alternative to MRI in appropriately selected patients, especially the elderly and/or those unsuitable or with contraindications to MRI. Integration of specific imaging features—such as peri-areolar skin thickening, NAC enhancement, tumor nipple enhancement and tumor-to-nipple distance—can improve diagnostic confidence and guide surgical planning. Further prospective studies are needed to confirm these findings and establish standardized criteria for NAC assessment across imaging modalities.

## Supplementary information

Supplementary information

## Abbreviations

BC: Breast cancer
BI-RADS: Breast Imaging Reporting and Data System
CEM: Contrast-enhanced mammography
CC: Cranio-caudal
DCIS: Ductal carcinoma in situ
EUSOMA: European Society of Breast Cancer Specialists
MLO: Medio-lateral oblique
NAC: Nipple-areolar complex
TTN: Tumor-to-nipple
