# Supplementary material for: Contrast-enhanced mammography versus breast MRI in the preoperative evaluation of the nipple-areola complex: data from a real-world setting
Source: Eur Radiol. 2026 Feb 24;36(7):5630–44. doi: 10.1007/s00330-026-12375-0 (PMC13282234; doi:10.1007/s00330-026-12375-0)
Supplement: Supplementary file 1 — Supplementary information [file 330_2026_12375_MOESM1_ESM.pdf]

Contrast-Enhanced Mammography versus Breast MRI in the Preoperative Evaluation of the Nipple-Areola Complex: Data from a Real-World Setting

ELECTRONIC SUPPLEMENTARY MATERIAL

Supplementary Table 1. NAC Infiltration and Histological Subtypes in CEM and MRI group.

		CEM<br>*(n = 91)*	MRI<br>*(n = 104)*	P Value
**NAC positivity n (%)**		10 (11)	20 (19.2)	0.110
**Histotype n (%)**	DCIS	5 (50)	10 (50)	0.145
	IBC-SNT	2 (20)	4 (20)	
	ILC	2 (20)	2 (10)	
	Other	1 (10)	4 (20)	
**Presence of "in situ" component n (%)**	Yes	9 (90)	15 (75)	0.598
	No	1 (10)	5 (25)	

Note. — *DCIS* ductal carcinoma in situ*, IBC-NST* Invasive breast carcinoma non-special type, *ILC* invasive lobular carcinoma. *Other* (1 Paget in CEM group and 2 DCIS + Paget, 1 DCIS + Lobular in situ carcinoma, 1 ca. mucinous in MRI group). P values for comparisons all variables were obtained with χ2 test.

Supplementary Table 2 - Multivariable logistic regression model testing interaction between patients' clinic characteristics and imaging modality (CEM vs MRI) in predicting NAC involvement.

Variable	OR	95% CI	P value
Imaging modality (CEM vs MRI)	0.02	0-10.77	0.231
Age (CEM patients vs MRI patients)	0.98	0.91-1.06	0.613
Breast density (dense vs non dense)	0.41	0.10-1.70	0.219
Interaction (Age × Imaging modality)	1.08	0.98-1.18	0.122
Interaction (Breast density × Imaging modality)	1	0.14-7.08	0.999

Note. — OR Odds Ratio, *95% CI* confidence interval. P values for comparisons were obtained with χ2 test for all other variables.
